# Vitamin D Supplementation as Add-on Therapy in Multiple Sclerosis—Balance between Benefit and Risk?: A Commentary on Vitamin D Supplementation in Central Nervous System Demyelinating Disease—Enough Is Enough

**DOI:** 10.3390/ijms20061513

**Published:** 2019-03-26

**Authors:** Hans-Klaus Goischke

**Affiliations:** Hochwaldstrasse 2, D-97769 Bad Brückenau, Germany; hkem.goischke@t-online.de

**Keywords:** multiple sclerosis, vitamin D supplementation, hypercalcemia, IL-6, serum neurofilament light chain

## Commentary

The excellent publication by Häusler and Weber [1] on the problem of vitamin D (VD) add-on therapy in multiple sclerosis could improve patient safety if the current state of knowledge is quickly applied to daily clinical practice used to care for people with multiple sclerosis (PwMS). However, the cases described to prove the risk of hypercalcemia in vitamin D supplementation (VDS) are extreme exceptions and are not representative of the majority of patients in everyday practice. The publication by Fragoso et al. was cited as a warning sign of vitamin D (VD) intoxication, with VD supplementation averaging 87,000 IU/day, with a median of 100,000 IU/day [2]. A moderate add-on therapy with vitamin D (VD) doses of up to 10,000 IU/day hardly presents a risk of hypercalcemia [3]. Although it is known that VD increases the absorption of calcium from the intestines, this effect is generally subject to physiological regulation. At serum levels above 80 nmol/L, intestinal Ca absorption is not increased. There is a balance between net Ca absorption from the intestine and calcium excretion in the kidney. An excessive production of the active 1,25(OH)D_3_ results in an adequate production of the catabolic enzyme 24-hydroxylase (CYP24A1), thus largely avoiding VD toxicity [4]. A possible mechanism of VD toxicity could be based on changes in metabolism by displacement of 1,25(OH)D from its binding protein (DBD). If the DBD binding capacity is exceeded, e.g., caused by 25(OH)D itself, hypercalcemia could occur [3]. Another cause of VD hypervitaminosis may be low activity of the catabolic enzyme 1,25(OH)_2_D-24-hydroxylase cytochrome P450 (CYP24A1). Mutations in the CYP24A1 gene are associated with a partial or total loss of 24-hydroxylase activity, which may result in hypercalcemia [4]. 

Patients with biallelic and, in some instances, monoallelic mutations of the CYP24A1 gene have elevated serum calcium concentrations associated with elevated serum 1,25(OH)_2_D, suppressed parathyroid hormone (PTH) concentrations, hypercalciuria, nephrocalcinosis, nephrolithiasis, and on occasion, reduced bone density [5]. In very rare cases, an imbalance of Ca/phosphorus homeostasis can occur without VD hypervitaminosis, and in extremely rare cases extremely high VD levels cannot cause hypercalcemia. Because VD intoxication without hypervitaminosis has been verified in anecdotal reports, calcium excretion can be determined by a 24 h urine collection (hypercalciuria) as an option for confirmation [6]. Hypercalciuria generally occurs before the development of hypercalcemia in VD hypervitaminosis [5].

Carlberg and Haq describe the concept of the personal vitamin D response index [7]. The concept is based on the fact that vitamin D3 activates via its metabolite 1α,25-(OH)2D_3_ the transcription factor vitamin D receptor and thus has a direct effect on the epigenome and transcriptome of many human tissues and cell types. Individuals can be classified as high, mid and low responders to VD by measuring VD sensitive molecular parameters, such as changes in the epigenetic status and the respective transcription of genes of mobile immune cells from blood or the level of proteins or metabolites in serum [7].

The unphysiological administration of high VD doses in studies (supraphysiological amounts weekly/monthly), which leads to a peak in VD serum levels, is given little consideration. The question arises as to whether the daily administration of VD doses (between 10,000 and 14,000 IE/day) would not lead to VD overdose less frequently and optimise the effects on the immune system. The half-life of circulating vitamin D3 is between 12 and 25 h. Because of this short half-life, even large bolus vitamin D doses of 50,000 IU to 100,000 IU are cleared from the circulation within a week, making vitamin D basically undetectable in the circulation. A daily dose of VD leads to a sustained increase in circulating 25(OH)D, which reaches a steady level after 3–4 months, while an acute, interval or large bolus dose of VD leads to a variety of appearance and disappearance rates. VD bolus administration can have profound effects on clinical trial results due to the short circulating half-lives of intact VD [8].

The authors interpret the study by Naghavi et al. to mean that the supplementation of 50,000 IU VD every five days leads to an increased activation of the immune cells in MS patients (up-regulates IL-6 and IL-17A) [9]. Hashemi et al., however, controversially assessed the effect of vitamin D3 supplementation on proinflammatory interleukine-17A, IL-6 and antiinflammatory IL-10 cytokines. Their study at PwMS registered a down-regulation of IL-17A and IL-6 under the same study conditions (50,000 IE VD/week over an 8-week therapy duration). The mRNA expression level of IL-10 increased, which should be achieved for PwMS [10]. IL-6 is a pleiotropic cytokine with significant functions related to the regulation of the immune system, especially autoimmunity [11]. Another study under the same study conditions (50,000IE VD/week) found a lower concentration of IL-6 (and TNF-α) after supplementation [12].

Attaining an optimal VD-serum level in northern regions through nutrition and sun exposure (with latitudes > 42 degrees N from the equator) without VDS in PwMS is more likely hopeful thinking than reality, especially considering the seasonal variants of the 25-OH vitamin D level between November and March/April. Relapse rates were higher during this time of the year. Germany lies between the 48th and 54th latitudes. Over a period of about 5 months, the UVB portion of the sun’s rays in these regions does not reach the level required to produce sufficient vitamin D via the skin [13]. The discussion on the effective VD level will be intensified if the disease activity can be assessed in the future by determining the neurofilament light chains (sNFL) in serum. There is overwhelming evidence that the sNFL values act as real-time markers to reflect disease activity over the last 3 months, and uncover asymptomatic persistent axonal damage not visible in the cerebral MRI [14], and that high sNFL concentrations also provide information on future brain and spinal cord volume loss [15]. The number of Gd-enhancement lesions in MRI correlated with the level of sNFL values [14,16]. As high NFL concentrations in serum and cerebrospinal fluid (CSF) correlated with one another, and an inverse association was found between serum 25(OH)D and CSF-NFL levels [17], the sNFL level would be the “neurologist’s CRP” [18]. If the add-on therapy with VD decreases the NFL level in the following 3–6 months, this would be proof that the correct daily VD dose was titrated for the individual patient. Then the corresponding individual VD serum level would be the benchmark for long-term therapy in the sense of personalized medicine. The effectiveness of VD therapy depends on the relevant individual VD dose and daily intake. At a weekly dose of 20,000 IU VD in PwMS (relapsing remitting MS-RRMs) and disease-modifying drugs (DMD), Holmoy et al. did not achieve an overall effect on sNFL levels, but in a subgroup of patients not receiving DMD, sNFL decreased more than 30% [19]. Cortese et al. demonstrated that each 50 nmol/L increase in mean 25(OH)D levels in the first 2 years post clinical isolated syndrome (CIS) diagnosis was associated with 20% lower sNFL levels [20].

Future studies must include sNFL values after daily supplementation with different VD doses and in relation to VD serum levels. In order to provide PwMS with an easily accessible parameter for disease activity worldwide, researchers and clinicians must agree on a standardized test for measuring sNFL as quickly as possible.

## Conclusions

The occurrence of side effects in VD add-on-therapy in multiple sclerosis is specifically documented by case histories with extremely high VD doses, administered weekly or monthly, where high unphysiological VD serum levels are reached. Daily substitution without additional oral calcium doses could lead to better results. Due to the short half-lives of circulating VD, weekly or monthly doses may have a profound effect on the results of clinical trials. Of course, rare side effects to patient safety should certainly be known, even though the add-on therapy is generally safe and cheap. A benefit and risk assessment remains important. It is evident that vitamin D status plays an important role in modulating the degree of MS disease activity. In the future, VD supplementation could be optimised by determining the serum neurofilament light chain.
